# Analysis of Two Neuroanatomical Subtypes of Parkinson's Disease and Their Motor Progression Based on Semi‐Supervised Machine Learning

**DOI:** 10.1111/cns.70277

**Published:** 2025-02-15

**Authors:** Hao Zhou, Yuqing Wu, Shuoying Chen, Yi Xing, Jingru Ren, Weiguo Liu

**Affiliations:** ^1^ Department of Neurology The Affiliated Brain Hospital of Nanjing Medical University Nanjing China

**Keywords:** free water, motor progression, Parkinson's disease, semi‐supervised machine learning, subtypes, voxel‐based morphometry

## Abstract

**Background:**

The high heterogeneity of Parkinson's disease (PD) hinders personalized interventions. Brain structure reflects damage and neuroplasticity and is one of the biological bases of symptomatology. Subtyping PD in the framework of brain structure helps in the prediction of disease trajectories and optimizes treatment strategies.

**Methods:**

The study included a total of 283 *de novo* PD and 141 healthy controls (HC). Structural heterogeneity between PD and HC was compared, and patients were classified using Heterogeneity through Discriminative Analysis. Gray matter volume (GMV), clinical symptoms, and substantia nigra free water (SNFW) among all subtypes were compared. These subtypes were followed for an average of 2.5 years to monitor motor impairment.

**Results:**

Early PD patients possessed higher GMV heterogeneity than HC, and two subtypes based on GMV patterns were identified. Subtype 1 showed widespread GMV reductions, while subtype 2 had an increased volume in the basal ganglia and parts of the cortex. Subtype 1 had more severe motor and non‐motor symptoms, as well as higher posterior SNFW. The whole‐brain GMV in the PD group was negatively correlated with posterior SNFW; basal ganglia volume in subtype 1 was negatively correlated with Unified Parkinson's Disease Rating Scale (UPDRS)‐III scores, whereas no linear correlation was found in subtype 2. The UPDRS‐III progression rate was higher in subtype 1 than in subtype 2 (2.52 vs 0.92 points/year).

**Conclusion:**

The heterogeneity of PD patients reflected the changes in their brain structure. The identification of these changes helps the classification of patients into different subtypes, additionally supported by clinical manifestations and SNFW, with consequent benefits for clinical consultancy and precision medicine.

## Graphical abstract

Control groups (represented by green dots) and patients (represented by red dots) are separated by convex polygon decision boundaries. The solid line represents the classifier, the dashed line represents the boundary, and the red highlighted solid line portion represents the convex polygon used to separate the patient from the control group.

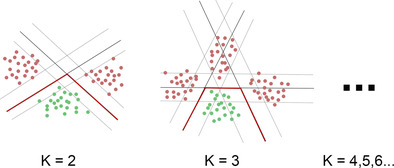

## 1 Introduction

Parkinson's disease (PD) is a highly heterogeneous neurodegenerative disorder characterized by a significant variability in clinical presentation and disease progression rate among patients [1]. To date, growing evidence shows that PD may be caused by distinct genetic or environmental factors that trigger the condition through different pathways only partially overlapping [2]. Subtyping has emerged as a methodological framework to better understand this heterogeneity by identifying distinct subgroups of PD patients, each characterized by specific features less commonly observed in other subgroups [3].

Most early subtype frameworks were based on predefined classification variables, such as age, genotype, dominance of tremor or rigidity, and the relative prevalence of motor versus non‐motor symptoms. The most widely used framework divided patients into two subtypes: (i) tremor dominant subtype, and (ii) postural instability and gait difficulties (PIGD) dominant subtype, based on the ratio of tremor scores to PIGD scores [4]. However, this approach is limited by the instability in the interconversion between subtypes [5] and the subjectivity associated with the selection of a cut‐off value for the tremor/PIGD score. Subsequently, Fereshtehnejad and colleagues identified three PD subtypes through a data‐driven approach using the Parkinson's Progression Markers Initiative (PPMI) cohort: mild motor‐dominant, diffuse malignant, and intermediate [6]. The classifier relies on a comprehensive and complex assessment framework (18 demographics and scales were included in the model) that requires a wide range of skills and technical abilities, and assessments may vary from physician to physician, increasing the difficulty of clinical practice. In addition to the PPMI database, subtype studies have also been performed in several Chinese cohorts. However, these studies are influenced by factors such as medication and disease stage, with a lack of longitudinal follow‐up [7, 8]. Therefore, the use of simple and objective classifiers (such as those based on neuroimaging) in an early, unmedicated PD cohort may be more reliable.

Structural magnetic resonance imaging (MRI) is a reliable in vivo imaging technique that provides highly stable images of brain structures, ideal for long‐term tracking and monitoring of neurodegenerative diseases such as PD, reflecting changes of the nerve damage and brain adaptation over the chronic course of the disease. Voxel‐Based Morphometry (VBM) is a structural imaging analysis method [9] extensively used to differentiate PD patients from healthy controls (HCs), to distinguish PD from atypical parkinsonian syndromes, and for subtyping PD [10]. A semi‐supervised machine learning technique known as Heterogeneity through Discriminant Analysis (HYDRA) [11] was used to address the structural heterogeneity of PD and identify neuroanatomical subtypes in drug‐naive PD patients. The strength of HYDRA lies in its dual ability to classify and cluster: it separates patients from HCs using a polyhedron formed by linear maximum margin classifiers and subsequently clusters patients according to their distance from various hyperplanes of the polyhedron. This approach reduces the impact of individual variations unrelated to the disease, facilitating the identification of genuine disease subtypes. To date, HYDRA has been successfully applied in research on Alzheimer's disease [11], schizophrenia [12], autism spectrum disorders [13], and obsessive‐compulsive disorder [14]. Our speculation is that it can discover different neuroanatomical subtypes of PD. Additionally, the free‐water values in the substantia nigra (SN) among different subtypes were compared to explore the potential pathological mechanisms behind these structural differences. Previous studies showed that increased free‐water values in the posterior substantia nigra (PSN) in PD are indicators of neuroinflammation and damage of the dopamine system, and are used to monitor disease progression [15, 16]. Moreover, a linear mixed‐effects model was used to predict the trajectory of motor impairment across the identified PD subtypes, hypothesizing that each neuroanatomical subtype is associated with a distinct clinical course.

## 2 Materials and Methods

### 2.1 Participants

*De novo* PD patients were recruited from a large PD cohort at the department of neurology, the Affiliated Brain Hospital of Nanjing Medical University, from 2018 to 2023. The inclusion criteria were the following: (1) diagnosis of PD by an experienced movement disorder specialist according to the Movement Disorder Society (MDS) diagnostic criteria [17]; (2) Hoehn & Yahr stage ≤ 3. The exclusion criteria were the following: (1) use of anti‐Parkinson's drugs at the time of recruitment; (2) diagnosis of atypical or secondary parkinsonian syndromes during follow‐up (follow‐up of at least one year by telephone or outpatient visits to monitor drug responses); (3) presence of metal implants in the body that preclude MRI examination, or significant brain abnormalities revealed by MRI scan; (4) severe chronic diseases such as heart failure, renal failure, or liver dysfunction; (5) meeting MDS diagnostic criteria for PD dementia [18]. A total of 288 PD patients who met the inclusion criteria were enrolled and completed a comprehensive assessment of the baseline clinical data; 90 patients underwent at least one in‐person follow‐up and complete follow‐up data were obtained. A total of 145 age‐, sex‐, and education‐matched HCs were recruited from a community in Nanjing and the health examination center of the affiliated brain hospital of Nanjing Medical University. Finally, five patients and four HCs were excluded due to poor MRI scan quality.

This study was approved by the Medical Ethics Committee of the Affiliated Brain Hospital of Nanjing Medical University. All participants provided written informed consent.

### 2.2 Clinical Evaluation

Detailed demographic and clinical data were collected through face‐to‐face interviews. The Unified Parkinson's Disease Rating Scale (UPDRS) part II and III and the modified Hoehn and Yahr (H‐Y) scale were used to assess daily living activities, motor impairment, and disease severity. The Montreal Cognitive Assessment (MoCA) was used to measure cognitive abilities. The Hamilton Depression (HAMD) scale and Hamilton Anxiety (HAMA) scale were used to assess the mental state of the patients. The Non‐Motor Symptoms Questionnaire (NMSQ) was used to evaluate the non‐motor symptoms. The levodopa equivalent daily dose (LEDD) was calculated during the follow‐up visits using a previously established method [19]. Some patients (37/90) were assessed using the MDS‐UPDRS scale during the follow‐up, and MDS‐UPDRS‐III was converted to the UPDRS‐III total score according to a previous study [20] to maintain consistency in the assessment before and after the follow‐up (see S1).

### 2.3 MRI Data Acquisition and Preprocessing

All T1‐weighted and DTI MRI scans were performed using a 3.0T Siemens Verio machine at the Affiliated Brain Hospital of Nanjing Medical University. The details of MRI scanning parameters are listed in S1.

The preprocessing and analysis of T1‐weighted images were performed using VBM in the Computational Anatomy Toolbox (CAT12.8.2; https://neuro‐jena.github.io/cat). All preprocessing steps use the default parameters of CAT12. The image quality was evaluated using an automated weighted average rating (IQR). A cut‐off value ≥ 75% was applied to ensure that the image quality was acceptable. Then, the regions of interest (ROIs) of the GMV were calculated according to the predefined automated anatomical labeling (AAL) atlas [21]. The GMV images were then smoothed using an isotropic Gaussian kernel with a full width at half maximum of 8 mm. DTI images were preprocessed using the MRtrix3 software (https://www.mrtrix.org/). The preprocessing steps included denoising, the removal of Gibbs ringing artifact, and the correction of motion and eddy current. Subsequently, scripts provided by the MarkVCID project (https://markvcid.partners.org/MarkvCID1‐Protocol‐Resources) were used to estimate the free water model. The detailed preprocessing information is listed in S1.

### 2.4 Heterogeneity and Between Group Differences in GMV

The potential greater inter‐individual variability of PD patients compared to HC was assessed. The individual differences were assessed by the extraction of the GMV for each region using the AAL atlas, resulting in a 1 × 116 GMV vector per subject. Then, the Euclidean distance between these vectors was calculated to create an *N* × *N* structural matrix for each group. The variability rate for each subject was defined as the mean of the *N*‐1 (excluding that subject) distance values, indicating their distance from other subjects in the group. This variability rate was computed separately for both the PD and HC groups.

### 2.5 Subtyping PD With HYDRA

The GMV was obtained from structural MRI data for subtyping from 116 AAL‐defined ROIs, using sex, age, years of education, and total intracranial volume (TIV) as covariates. HYDRA (https://github.com/evarol/HYDRA) identifies distinct subtype clusters of *de novo* PD through the volumetric measurement of ROIs. It determines the number of hyperplanes according to K (number of clusters), creating convex polyhedra to differentiate PD patients from HCs. The distance of each patient to each hyperplane was calculated with an extended standard linear maximum margin classifier, assigning patients to their nearest hyperplane and defining K clusters. The clustering range was set between 2 and 6, and the following parameters were also set: 50 iterations between estimating hyperplanes and cluster estimation, 20 clustering consensus steps, 0.25 regularization parameter, and 10 cross‐validation folds. This method uses the Adjusted Rand Index (ARI) to assess clustering stability [22] that ranges from 0 to 1. Ideally, the ARI is the highest for the best K value and lower for other K values.

### 2.6 Voxel‐Wise Volumetric Analysis of PD Subtypes

A two‐sample t‐test was used to compare GMV between HC and two subtypes according to age, sex, education, and TIV. Statistical analysis was performed using Data Processing & Analysis for Brain Imaging (DPABI V7.0, http://rfmri.org/DPABI) [23], and multiple comparisons were corrected using Gaussian Random Field theory (GRF) (voxel *p* < 0.001, cluster *p* < 0.01).

### 2.7 Reproducibility Analysis of PD Subtypes

A split‐sample analysis approach, which is often used in cluster analysis, was used to investigate the reproducibility of PD subtypes [12, 13]. PD patients and HC were randomly divided into two equal groups (split 1 and split 2). The HYDRA algorithm was applied to each split, and GMV differences between subtypes and HC were compared within each group.

### 2.8 Free Water Value Extraction

The b0 images were registered and normalized to MNI space. Subsequently, two raters manually delineated ROIs for the anterior substantia nigra (ASN) and posterior substantia nigra (PSN) using the ITK‐SNAP toolbox (http://www.itksnap.org). Two ROIs (each 2 × 2 mm per slice, covering 8 voxels) were placed on each side of the SN across two consecutive slices according to previous research [15]. The process involved the identification of the slice with the largest red nucleus volume (S0), moving ventrally to the slice where the red nucleus faintly disappeared (S1), and then to the slice where it was invisible (S2). ROIs were placed on S1 and S2 at the darkest regions corresponding to ASN and PSN. The intraclass correlation coefficient (ICC) was calculated to assess the consistency between raters, and the primary rater's ROIs were used for the final analysis. Then, average bilateral ROIs were used to obtain the final ASN and PSN free water values.

### 2.9 Statistical Analysis

Statistical analysis was performed using IBM SPSS Statistics version 27.0 or R version 4.4.0 (https://www.r‐project.org/). Continuous variables were described as mean ± standard deviation. The Kolmogorov–Smirnov test was used to assess the normality of continuous variables. The two‐sample *t*‐test was used to compare normally distributed data; otherwise, the Mann–Whitney U test was used to compare continuous variables between the two subtypes. Categorical variables were presented as percentages, and the Chi‐square test was used to compare group differences. A general linear model compared free water values (ASN and PSN) between subtypes, adjusting for age, sex, education, TIV, and disease duration. The same model was applied to compare PD subgroups and HC, adjusting for age, sex, education, and TIV. Partial correlation analysis was used in the identified PD subtypes to examine the correlation between the GMV of basal ganglia nuclei (including the caudate nucleus, putamen, and globus pallidus) and UPDRS‐III scores, using age, sex, years of education, TIV, and disease duration as covariates. Partial correlation analysis performed in PD patients was used to examine the correlation between PSN free water values and GMV, using age, sex, years of education, TIV, and disease duration as covariates.

A follow‐up of at least one year after the baseline assessment was performed on 90 patients, and it was completed on‐site, providing relatively complete clinical data. The association between different PD subtypes and the longitudinal progression of the disease was assessed using a linear mixed‐effects model. This progression was evaluated by repeatedly measuring UPDRS‐III scores at different time points. Linear mixed‐effects models are used in the presence of data imbalance due to missing data, patient dropout, non‐uniform follow‐up intervals, and variable initial assessment times, taking into account the correlation among repeated measures on individuals, making it ideal for analyzing the trajectories of longitudinal markers [24]. Time, PD subtype, and their interaction effect were included in the fixed effects part of the model, while using sex, age at disease onset, and LEDD at follow‐up as covariates to construct the predictive model of disease progression. Additionally, the model included random intercepts for patient ID and random slopes for time to better adjust for individual differences. The model generated predicted values for each observation, and predicted disease progression trajectories were plotted using ggplot. In the model, two‐tailed value of *p* < 0.05 was considered statistically significant.

## 3 Results

### 3.1 Higher Heterogeneity of GMV in PD

**FIGURE 1:**
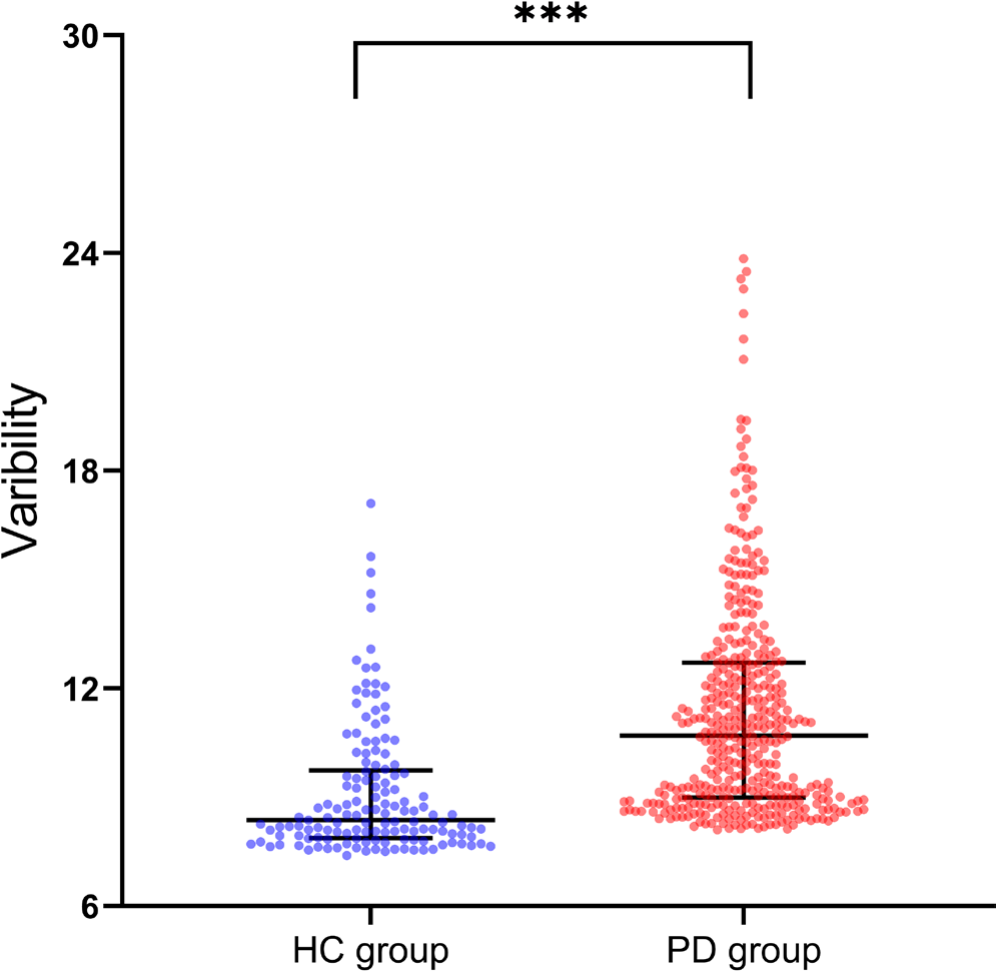
Variability analysis. PD patients showed higher structural heterogeneity than HC. PD, Parkinson's disease; HC, healthy control.

The variability for each individual in the PD and HC groups was calculated separately to discover the heterogeneity in GMV. The variability in PD patients was significantly higher than that in the HC group (*p* < 0.001, Figure 1).

### 3.2 HYDRA Reveals Two PD Subtypes

**FIGURE 2:**
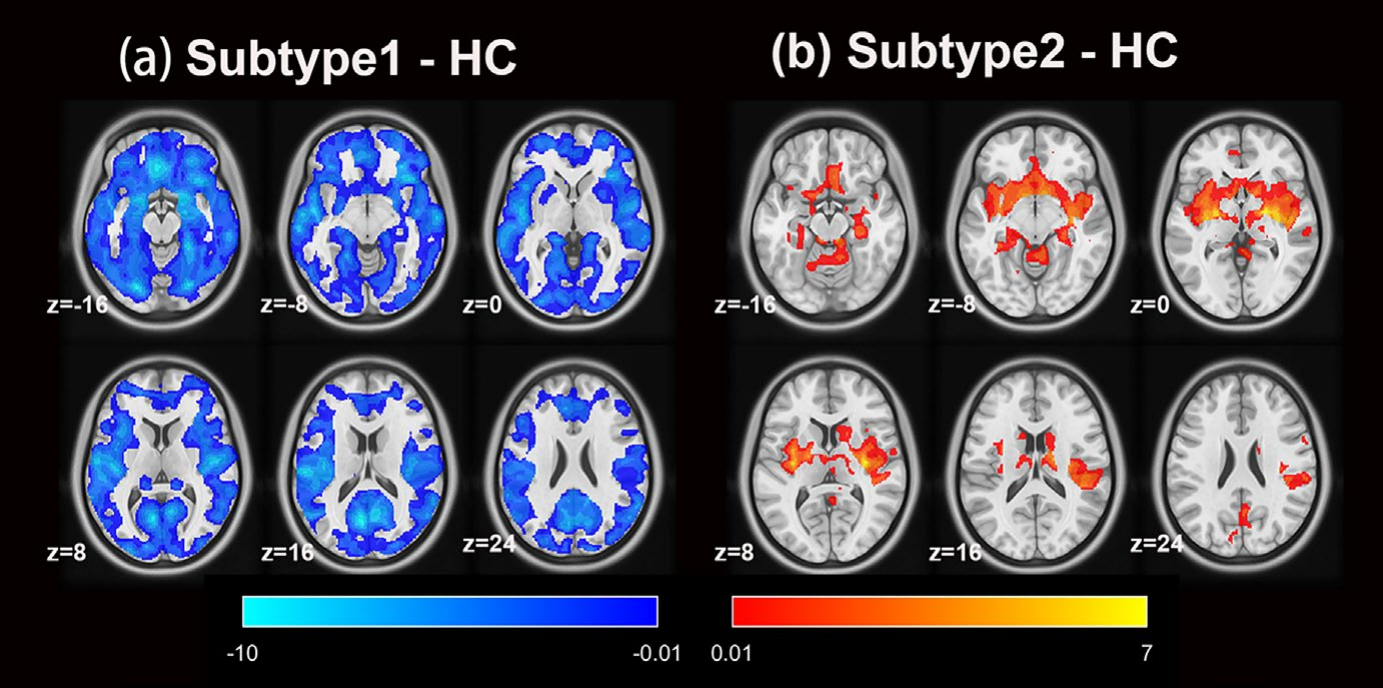
Patterns of gray matter volumes in the two PD subtypes. (a) PD‐subtype1 showed widespread loss of gray matter volumes compared to HCs. (b) PD‐subtype2 showed regional increased gray matter volumes compared to HCs. Gaussian Random Field theory correction (voxel *p* < 0.001, cluster *p* < 0.01).

The most stable classification results were achieved when *K* = 2, with an ARI of 0.825 (Figure S1). Subtype 1 showed a widespread reduction in GMV across the whole brain compared to HC (Figure 2a). Subtype 2 showed an increased volume in the subcortical nuclei, including the bilateral caudate nuclei, amygdalae, putamen, globus pallidus, and thalamus. Furthermore, localized increases in cortical volume were found, notably in the medial bilateral frontal lobes, bilateral insular cortices, bilateral parietal lobes, and certain regions of the cerebellum (Figure 2b).

### 3.3 Reproducibility Results

Both split 1 and split 2 achieved the highest ARI at K = 2, with ARI values of 0.844 and 0.772, respectively (Figure S2). VBM analysis showed that the two split samples possessed similar differential brain regions compared to the complete sample (Figure S3). Therefore, our subtype classification results were reliable and reproducible.

### 3.4 Clinical Examination of PD Subtypes

**FIGURE 3:**
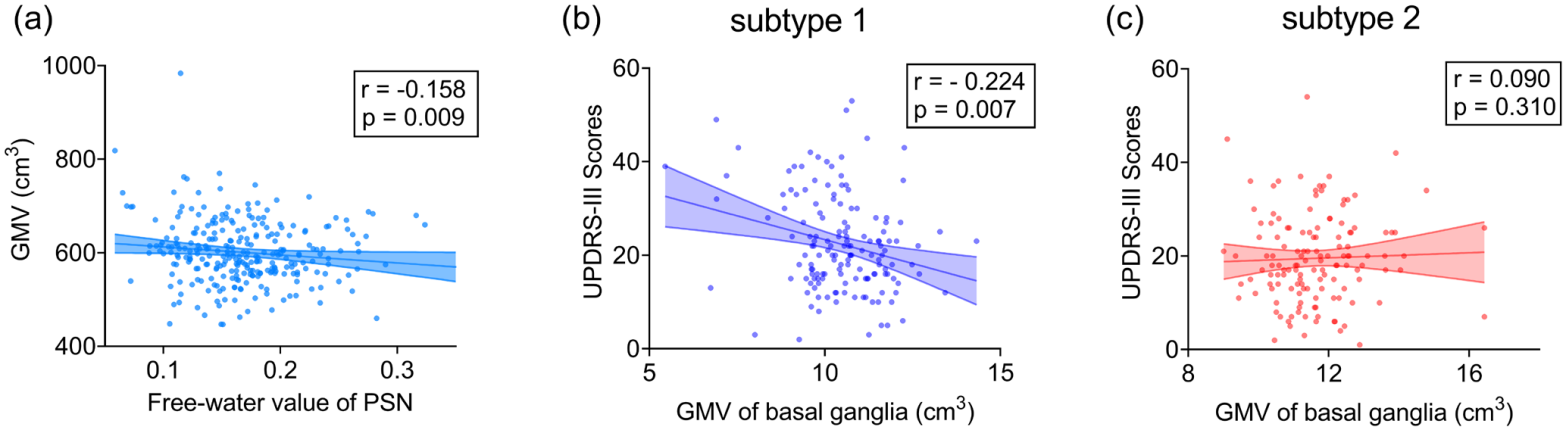
Correlation analysis (a) in PD patients; whole brain GMV was negatively correlated with PSNFW. (b) Subtype 1 basal ganglia GMV and UPDRS‐III scores were negatively correlated. (c) No linear correlation was found in subtype 2.

A total of 149 cases were included in subtype 1 and 134 in subtype 2. Clinically, subtype 1 showed more severe motor symptoms (UPDRS‐III, 22.4 ± 10.3 vs 19.5 ± 9.5, *p* = 0.031) and more severe non‐motor symptoms (NMSQ, 8.2 ± 4.5 vs 6.9 ± 4.2, *p* = 0.017) compared to subtype 2. Furthermore, subtype 1 showed a moderate cognitive impairment (MoCA, 21.2 ± 5.3 vs 22.3 ± 4.9, *p* = 0.065) compared to subtype 2. The free water value in the PSN of subtype 1 was significantly higher than that of subtype 2 (0.176 ± 0.047 vs 0.160 ± 0.048, *p* = 0.008) (Table 1). The ICC analysis demonstrated a good consistency across all SN ROIs. The ICC value was 0.841 for the right ASN, 0.800 for the left ASN, 0.919 for the right PSN, and 0.900 for the left PSN. PSN free water values in PD patients were negatively correlated with whole‐brain GMV (*r* = −0.158, *p* = 0.009) (Figure 3a). The basal ganglia GMV in subtype 1 patients showed a negative correlation with UPDRS‐III scores (*r* = −0.224, *p* = 0.007), while no linear correlation was observed in subtype 2 patients (Figure 3b,c).

**TABLE 1:** Demographic and clinical characteristics among subgroups.

item	PD‐subtype 1 (*n* = 149)	PD‐subtype 2 (*n* = 134)	HCs (*n* = 141)	*p* value		
				type 1 vs. type 2	type 1 vs. HC	type 2 vs. HC
Age	59.6 ± 8.9	59.6 ± 7.8	60.2 ± 7.2	0.973^a^	0.516^a^	0.473^a^
Male (%)	71 (47.7)	62 (46.3)	64 (45.4)	0.905^b^	0.725^b^	0.904^b^
Education	9.4 ± 4.3	9.7 ± 4.3	9.9 ± 3.8	0.499^a^	0.268^a^	0.705^a^
Age at onset	57.9 ± 9.0	57.7 ± 7.9	NA	0.882^a^	—	—
Disease duration	1.7 ± 1.3	1.9 ± 1.4	NA	0.472^a^	—	—
TIV, cm^3^	1434 ± 144	1458 ± 164	1396 ± 154	0.198^a^	**0.029** ^**a**^	**0.001** ^**a**^
UPDRS–II	7.4 ± 3.5	6.8 ± 3.8	NA	0.131^c^	—	—
UPDRS‐III	22.4 ± 10.3	19.5 ± 9.5	NA	**0.031** ^**c**^	**—**	**—**
H‐Y stage (1/1.5/2/2.5/3)	(38/34/51/22/4)	(54/26/38/12/4)	NA	0.093^b^	—	—
MoCA	21.2 ± 5.3	22.3 ± 4.9	26.0 ± 4.0	0.065^c^	**< 0.001** ^**c**^	**< 0.001** ^**c**^
NMSQ	8.2 ± 4.5	6.9 ± 4.2	NA	**0.017** ^**c**^	**—**	**—**
HAMD	9.7 ± 7.0	9.0 ± 6.5	NA	0.339^c^	—	—
HAMA	6.9 ± 5.6	6.1 ± 4.4	NA	0.398^c^	—	—
Free water of ASN	0.118 ± 0.038	0.109 ± 0.042	0.095 ± 0.042	0.138^d^	**< 0.001** ^**e**^	**0.023** ^**e**^
Free water of PSN	0.176 ± 0.047	0.160 ± 0.048	0.139 ± 0.049	**0.008** ^**d**^	**< 0.001** ^**e**^	**0.004** ^**e**^

*Note:* Significant values are in bold (*p* < 0.05).

^a^ Two‐sample *t*‐test.

^b^ Chi‐squared test.

^c^ Mann‐Whitney U test.

^d^ General linear model, adjusted for age, sex, education, TIV, and disease duration.

^e^ General linear model, adjusted for age, sex, education, and TIV.

Abbreviations: ASN, anterior substantia nigra; HAMA, Hamilton Anxiety Scale; HAMD, Hamilton Depression Scale; H–Y Stage, Hoehn & Yahr Stage; MoCA, Montreal Cognitive Assessment; NMSQ, Non‐Motor Symptoms Questionnaire; PD, Parkinson's disease; PSN, posterior substantia nigra; TIV, total intracranial volume; UPDRS, Unified Parkinson's Disease Rating Scale.

### 3.5 Results of Linear Mixed Effects Models

**FIGURE 4:**
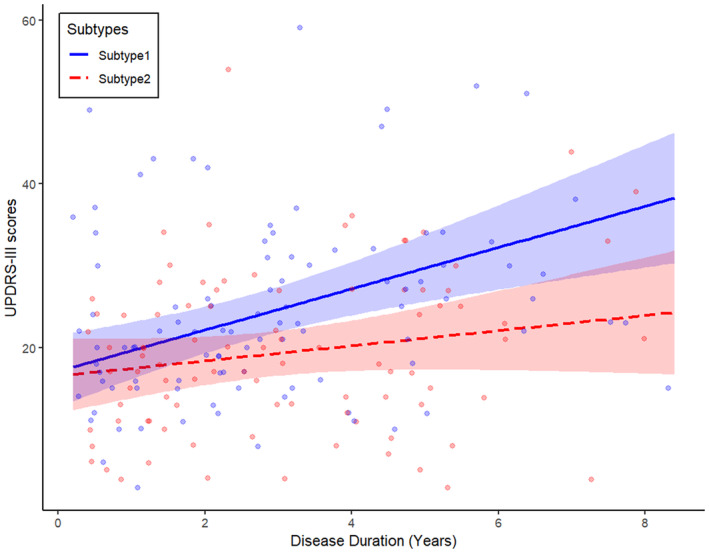
Longitudinal predictive trajectory of the Uniform Parkinson's Disease Scoring Scale Motor Score (UPDRS‐III) for two Parkinson's disease subtypes.

The progression rate (standard error, SE) of UPDRS‐III scores in subtype 1 was higher than that in subtype 2 [2.52 (0.66) vs 0.92 (0.65) points/year], with a difference of 1.60 (0.73) points/year (*p* = 0.031) (Table 2 and Figure 4). The average follow‐up time was 2.3 ± 1.0 years for subtype 1 and 2.8 ± 1.2 years for subtype 2. Patients' information used in the linear mixed‐effects model is listed in Table S1.

**TABLE 2:** Association between two subtypes and annual change in Unified Parkinson's Disease Rating Scale (UPDRS) motor scores using linear mixed‐effects models.

Main effect	*β* (SE)	t value	*p* value
Intercept	13.12 (7.10)	1.849	0.068
Group	−0.58 (2.75)	−0.210	0.834
Time	2.52 (0.66)	3.816	**< 0.001**
Sex	1.05 (2.00)	0.527	0.600
LEDD at visit	−0.0044 (0.0035)	−1.234	0.219
Age at onset	0.06 (0.12)	0.470	0.640
Group interaction with time	−1.60 (0.73)	−2.203	**0.031**

*Note:* Model adjusted for sex, age at onset, and LEDD at visit. The main effect of group indicates the effect of two subtypes on the intercept, while the interaction with time indicates the effect of two subtypes on the slope (annual change in UPDRS motor scores). Significant values are in bold (*p* < 0.05).

Abbreviations: LEDD, levodopa equivalent daily dose; SE, standard error.

## 4 Discussion

In this study, a data‐driven clustering method without prior assumptions was used to identify two robust neuroanatomical subtypes among drug‐naive PD patients with similar demographic characteristics in a large Chinese cohort. Several key results were found: first, the cross‐sectional data revealed the following evidence: (1) PD exhibited significant structural heterogeneity, with subtle morphological changes detectable in the early stages by VBM analysis; (2) GMV served as a basis for PD subtyping, with high‐volume patients displaying milder symptoms and slower disease progression in the early stage, potentially caused by brain functional compensation; (3) GMV was correlated with PSN free water content, suggesting that volume changes might be linked to neuroinflammation. Both GMV and PSN free water effectively reflected the imaging characteristics of early PD and possessed the potential for monitoring disease progression. Second, the longitudinal data were consistent with the cross‐sectional findings, indicating that HYDRA was advantageous in identifying PD structural heterogeneity. These results contributed to understanding the complex pathology of PD and predicting clinical outcomes.

Our results provided insights into the structural heterogeneity of PD. Although various factors such as education level, geographic location, genetic background, and socioeconomic status might influence brain development in the general population [25, 26, 27], the variation observed in PD patients significantly exceeded that observed in HCs. Patients in subtype 1 showed more extensive neuroanatomical changes, affecting almost the entire brain compared to HCs, while subtype 2 patients showed changes primarily in the basal ganglia and limbic system. This difference might be due to variations in the deposition sites, spread rate, and neuroadaptive changes of alpha‐synuclein among individuals [28].

Moreover, our study might help the understanding of the differences in previous research findings regarding GMV changes in PD patients. As a neurodegenerative disease, a reduced GMV is expected in PD patients, since the abnormal folding and deposition of alpha‐synuclein can lead to selective and progressive neuronal death through mechanisms such as mitochondrial damage, lysosomal dysfunction, and impaired calcium homeostasis [29]. Most previous studies reported regional or widespread GMV reduction in PD patients [30]. The nigrostriatal damage is the core pathology of PD. Geng et al. found that the putamen volume in patients with early‐ and late‐stage PD is reduced by 12.5% and 26.5%, respectively, and that putamen volume is negatively correlated with H‐Y stage [31]. Furthermore, Li et al. discovered that PD patients possess an atrophic caudate nucleus, with GMV loss gradually extending from the basal ganglia to the angular gyrus and temporal lobe as PD progresses, eventually spreading through the subcortical–cortical network [32]. Besides the putamen and caudate nucleus, few studies are available on the morphological changes in the pallidum. However, as one of the nuclei regulating movement, it is likely affected in PD. Menke et al. found morphological changes in the right pallidum in patients with early‐stage PD using sensitive shape analysis [33]. A study also reported that pallidum atrophy is more pronounced in late‐stage PD [31]. Our results showed significant basal ganglia GMV atrophy in subtype 1 patients, closely associated with disease severity, consistent with the striatal damage characteristic of PD. This suggested that basal ganglia GMV could be an effective indicator for monitoring neurodegenerative changes in this subtype of patients.

In contrast, regional increases in GMV require careful interpretation. Brain volume may serve as a critical mediator of brain reserve, helping the promotion of the recovery of large‐scale brain networks and the maintenance of normal function in the face of PD and other neurodegenerative pathologies [34, 35, 36, 37]. Besides, behavioral training can enhance brain reserve by increasing brain volume. Sehm et al. revealed that PD patients experience an increase in cerebellar GMV after balance training, accompanied by significant clinical behavioral improvements [38], suggesting a positive impact on brain volume. Moreover, the compensatory increase in brain volume can effectively counteract early motor impairments when faced with pathological damage. Reetz et al. showed that asymptomatic carriers of the Parkin gene have increased GMV in specific regions of the basal ganglia, suggesting that presynaptic dopamine dysfunction initially leads to neuronal hyperactivity and chronic hypertrophy, which is a result of striatal re‐adaptation that compensates for dopaminergic deficits in motor circuits even after the onset of symptoms [39]. Wang et al. conducted a clustering analysis in 2020 on the PPMI cohort, identifying two distinct subtypes based on subcortical volume patterns [36]. The low‐volume group has more severe baseline symptoms, faster progression across multiple disease dimensions during the follow‐up, and poorer prognosis than the high‐volume group. They explained this phenomenon through the possibility that the high‐volume group possesses more neurons and synapses to maintain brain function, whereas the low‐volume group lacks the corresponding compensatory capacity. Different individuals may experience various degrees of neurodegeneration and adaptive changes in specific cortical and basal ganglia regions due to the progressive development of PD symptoms and the long prodromal phase. Furthermore, subtype 1 patients showed significantly higher free water value in the PSN than that in subtype 2. Although increased free water does not directly indicate the loss of dopaminergic neurons, it does reflect neurodegenerative changes such as atrophy, disruption of the blood–brain barrier, and neuroinflammatory responses [16]. In a multicenter study, PSN was associated with H‐Y staging, MDS‐UPDRS‐III, and cognitive ability [40], and longitudinal data showed that increasing PSN over time predicts the severity of bradykinesia and cognitive decline [41], which was confirmed in a larger study [15]. The correlation analysis in our study showed that the free water content in the PSN was negatively correlated with GMV, suggesting that atrophy in brain volume might be associated with neuroinflammation.

In our cohort, the baseline data were consistent with the follow‐up data, indicating that the subtype with milder baseline symptoms experienced slower disease progression in the early stages. GMV increased in subtype 2, where it was primarily concentrated in the basal ganglia, insula, medial frontal lobe, parietal lobe, and parts of the cerebellum. These regions significantly overlapped with motor reserve networks described in previous reports [42]. Increases in gray matter volume in specific regions may provide stronger motor reserves to patients, leading to milder motor symptoms at baseline and slower progression. Jeong et al. used a linear mixed‐effects model to assess the impact of striatal volume associated with motor reserve on the longitudinal increase in LEDD during the follow‐up [43]. They found that patients with larger striatal volumes require less LEDD in the early stages. In our study, both PD subtypes received similar doses of levodopa replacement therapy (approximately 400 mg/day). Subtype 2 patients showed stable symptoms in the early stage of the disease (≤ 5 years), which also reflected the lower early LEDD requirement in patients with higher striatal GMV.

The strength of this study is our patient population, which comes from an early untreated cohort, allowing us to classify PD patients and predict disease progression as early as possible, without the influence of medication. This is useful for providing clinical advice, care, personalized treatment, and further cohort studies. Specifically, patients with subtype 1 are advised to increase the number of visits and the frequency of drug therapy adjustments, along with integrated management of more severe non‐motor symptoms and neuroinflammation. For example, take melatonin and TMS intervention to improve sleep quality [44, 45] and exercise, diet intervention to improve neuroinflammation [46, 47], and psychological intervention to improve patients' chronic psychological stress caused by the disease. In addition to providing more aggressive treatment options for patients with subtype 1 in clinical practice, subtype 1 may be a more suitable target for designing clinical drug trials. In the context of monoclonal antibodies, both Prasinezumab and Cinpanemab did not meet the primary endpoint [48, 49]. Nevertheless, exploratory analyses indicated that Prasinezumab is effective in delaying motor progression among patients receiving MAOB inhibitors and those exhibiting rapid disease progression [50]. This highlights the importance of patient‐stratified management. According to the findings of this study, the use of MAOB inhibitors (with potential neuroprotective effects) [51] should be prioritized for subtype 1 patients. Additionally, clinical trials should preferentially include this patient group to more accurately capture treatment effects. As regards subtype 2, maintaining low‐dose anti‐PD therapy while achieving satisfactory efficacy might help reduce drug‐related complications.

This study also has the following limitations: (1) despite the two neuroanatomical subtypes differing in symptom severity and disease progression at the group level, an overlap at the individual level was observed. Since GMV increases and decreases are continuous processes, the binary classification at the group level could not finely capture the changes. The use of continuous membership probability values in larger cohorts in the future could help in understanding better the differences in neuroanatomical subtypes. (2) Our cohort included only early‐stage PD patients, with an average disease duration of less than 2 years at baseline. The average total disease length was less than 5 years even during follow‐up, preventing the collection of late‐stage disease characteristics. Longer follow‐up periods are needed in the future to clarify long‐term prognosis. (3) All patients were evaluated using UPDRS at baseline. Some patients (37/90) were evaluated using MDS‐UPDRS during follow‐up. The differences between these scales limited the comparison of subitems. (4) Several critical factors related to clinical phenotypes, such as olfactory function, autonomic symptoms, and REM sleep behavior disorder, were not included in the current analysis, thus limiting the comprehensiveness of our subtyping description. Our future investigations will include these elements to enhance our evaluation framework.

## 5 Conclusion

A sophisticated semi‐supervised machine learning method was used to robustly identify two distinct neuroanatomical subtypes in a Chinese cohort of newly diagnosed PD patients. These subtypes showed different patterns of GMV, clinical symptoms, substantia nigra free water values, and disease progression rates. The recognition of the anatomical heterogeneity of PD is useful for understanding the complex pathology of PD, predicting clinical outcomes, and exploring potential biomarkers. This knowledge could be used to improve clinical decision‐making and optimize treatment strategies.

## Ethics Statement

The Medical Ethics Committee of the Affiliated Brain Hospital of Nanjing Medical University approved the study protocol.

## Conflicts of Interest

The authors declare no conflicts of interest.

## Supporting information


Data S1.

## Data Availability

The data that support the findings of this study are available from the corresponding author upon reasonable request.
