# Supplementary material for: Analysis of Two Neuroanatomical Subtypes of Parkinson's Disease and Their Motor Progression Based on Semi‐Supervised Machine Learning
Source: CNS Neurosci Ther. 2025 Feb 15;31(2):e70277. doi: 10.1111/cns.70277 (PMC11829112; doi:10.1111/cns.70277)
Supplement: Supplementary file 1 — Data S1. [file CNS-31-e70277-s001.docx]

**Analysis of two neuroanatomical subtypes of Parkinson's disease and their motor progression based on semi-supervised machine learning**

**Supplementary Materials**

Materials and methods

Supplementary tables:

Table S1

Supplementary figures:

Figure S1, Figure S2, Figure S3

**Materials and methods**

**MRI data acquisition and processing**

T1-weighted images were acquired using a coronal 3D gradient echo sequence with the following settings: repetition time (TR) of 2530 ms, echo time (TE) of 3.34 ms, flip angle of 7 degrees, 128 slices, slice thickness of 1.33 mm with a 0.5 mm gap, acquisition matrix of 256×192, and a field of view (FOV) of 256 mm×256 mm. DTI data were obtained using a spin-echo planar imaging sequence on an axial plane parallel to the anterior-posterior commissure line with the following parameters: TR of 9000 ms, TE of 104 ms, flip angle of 7 degrees, FOV of 230 mm×230 mm, slice thickness of 2.5 mm with no gap, matrix size of 128×128, 49 slices covering the entire brain, a b-value of 0 and 1000 s/mm², and 64 diffusion gradient directions.

The preprocessing and analysis of T1-weighted images were performed using VBM within the Computational Anatomy Toolbox (CAT12.8.2; <https://neuro-jena.github.io/cat>). The primary steps included normalizing the T1 images to the Montreal Neurological Institute (MNI) space, resampling the voxel size to 1.5×1.5×1.5 mm³, segmenting the normalized images into gray matter, white matter, and cerebrospinal fluid, and then converting the tissue densities’ voxel values to volumes. The quality of images was assessed by using the automated weighted average image quality rating (IQR) included in the CAT12 toolbox (http://neuro.uni-jena.de/ cat). This score represents a weighted average of local (noise contrast ratio) and global (inhomogeneity contrast ratio) standard deviations within the optimized white matter segment, scaled by the minimum tissue contrast and adjusted for voxel size through root mean square calculations. A PDF report is generated for all subjects, containing percentage ratings (0-100%) along with corresponding letter grades (F - A+) for noise, bias (inhomogeneity), resolution, and IQR. The obtained quality ratings categorize images into three groups: high-quality images (grades A+, A, B), moderate-quality images (grades C, D, E), and low-quality images (grade F indicating problematic scans). We applied a cut-off criterion of >=75% or >= grade C to ensure that only acceptable quality images were included in our analysis since low-quality scans can lead to underestimations of gray matter during preprocessing. The general IQR ratings observed in this study were as follows: 83.2% ± 2.6% (mean ± sd) in healthy controls (HC), 83.6% ± 2.6% in subtype 1, and 83.8% ± 2.8% in subtype 2; no significant differences were found among these three groups.

DTI images were preprocessed using the MRtrix3 software (<https://www.mrtrix.org/>). The preprocessing steps included denoising, Gibbs ringing artifact removal, motion correction, and eddy current correction. Subsequently, scripts provided by the MarkVCID project (<https://markvcid.partners.org/MarkvCID1-Protocol-Resources>) were employed to estimate the free water model. This model analyzes two distinct compartments: the free water compartment, which simulates the isotropic diffusion of water molecules in the extracellular space to highlight the situation of extracellular water molecules; and the tissue compartments, which reflect the intracellular microstructure after the free water signal has been removed. The fractional volume of the free water compartment, an index measuring the content of free water, shows the proportion of free water in each voxel, with values ranging from 0 to 1.

**Scale conversion**

Some patients (37/90) were assessed using the MDS-UPDRS scale during the follow-up, and MDS-UPDRS-III was converted to the UPDRS-III total score according to a previous study^1^ to maintain consistency in the assessment before and after the follow-up. Specifically, the UPDRS-III total score is calculated by subtracting 7 from the MDS-UPDRS-III total score. The proportion of MDS-UPDRS evaluated in subtype 1 and subtype 2 groups was 19/45 and 18/45, with no statistical difference by Chi-square test.

Table S1 Demographic and clinical characteristics of two PD Subtypes used for longitudinal follow-up.

| item | PD - Subtype1 (n = 45) | PD - Subtype2 (n = 45) | *P* value |
| --- | --- | --- | --- |
| Age, years ± SD | 57.6 ± 9.4 | 60.1 ± 7.3 | 0.163^a^ |
| Sex, male (%) | 23 (51.1) | 21 (46.7) | 0.833^b^ |
| Education, years ± SD | 10.1 ± 3.6 | 10.1 ± 4.2 | 0.936^a^ |
| Age at onset, years ± SD | 55.9 ± 9.5 | 58.4 ± 7.4 | 0.168^a^ |
| Disease duration, years ± SD | 1.7 ± 1.3 | 1.7 ± 1.1 | 0.957^a^ |
| TIV, mm^3^ ± SD | 1435 ± 139 | 1471 ± 174 | 0.289^a^ |
| UPDRS – II, n ± SD | 7.0 ± 2.6 | 6.8 ± 3.4 | 0.643^c^ |
| UPDRS – III, n ± SD | 21.1 ± 9.8 | 19.3 ± 9.8 | 0.383**^c^** |
| H-Y stage (1/1.5/2/2.5) | (13/11/17/4) | (19/12/12/2) | 0.437^b^ |
| MoCA, n ± SD | 22.2 ± 4.6 | 22.8 ± 4.7 | 0.402^c^ |
| NMSQ, n ± SD | 8.6 ± 5.0 | 6.8 ± 4.6 | 0.052**^c^** |
| HAMD, n ± SD | 10.9 ± 7.2 | 8.2 ± 5.6 | 0.069^c^ |
| HAMA, n ± SD | 7.7 ± 6.0 | 5.6 ± 4.3 | 0.124^c^ |
| Free water of ASN, n ± SD | 0.116 ± 0.041 | 0.107 ± 0.049 | 0.384^d^ |
| Free water of PSN, n ± SD | 0.185 ± 0.055 | 0.156 ± 0.050 | **0.023^d^** |
| LEDD at last visit, n ± SD | 363.1 ± 210.0 | 374.9 ± 259.4 | 0.813^a^ |

PD, Parkinson’s disease; SD, standard deviation; TIV, total intracranial volume; UPDRS, Unified Parkinson’s Disease Rating Scale; H-Y Stage, Hoehn & Yahr Stage; MoCA, Montreal Cognitive Assessment; NMSQ, Non-Motor Symptoms Questionnaire; HAMA, Hamilton Anxiety Scale; HAMD, Hamilton Depression Scale; ASN, anterior substantia nigra; PSN, posterior substantia nigra; LEDD, Levodopa Equivalent Daily Dose.

^a^Two-sample t-test, ^b^Chi-squared test, ^c^Mann-Whitney U test; ^d^Analysis of covariance, adjusted for age, sex, education, TIV and disease duration.

Significant values are in bold (*p* < 0.05).


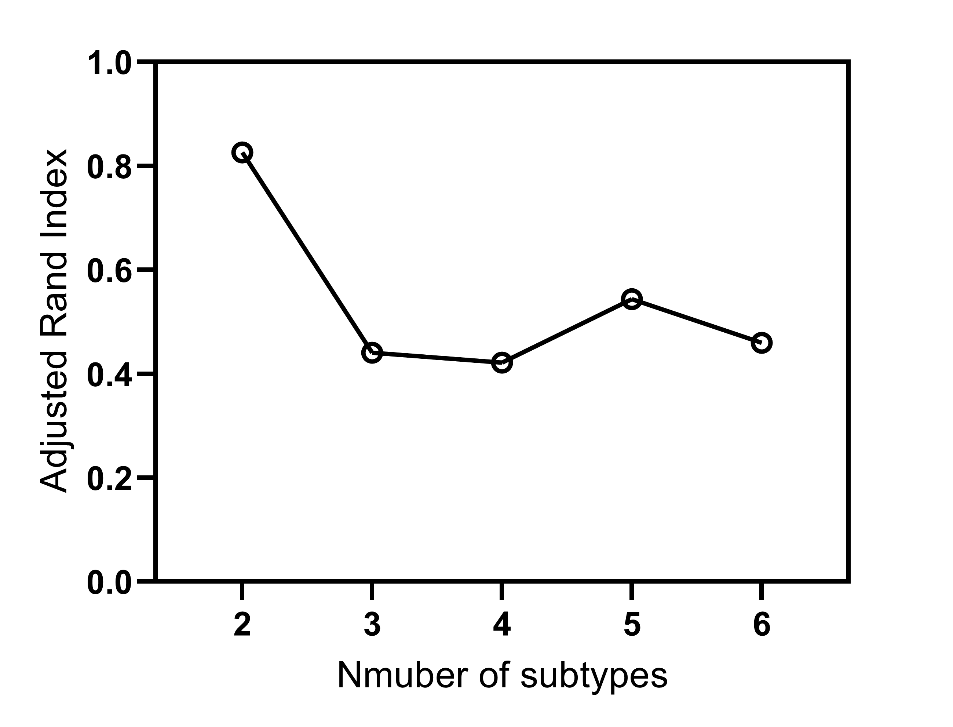


**Figure S1** Clustering stability. The adjusted rand index is used to evaluate the stability of the cluster, and results indicate that K = 2 achieves highest reproducible subtypes.


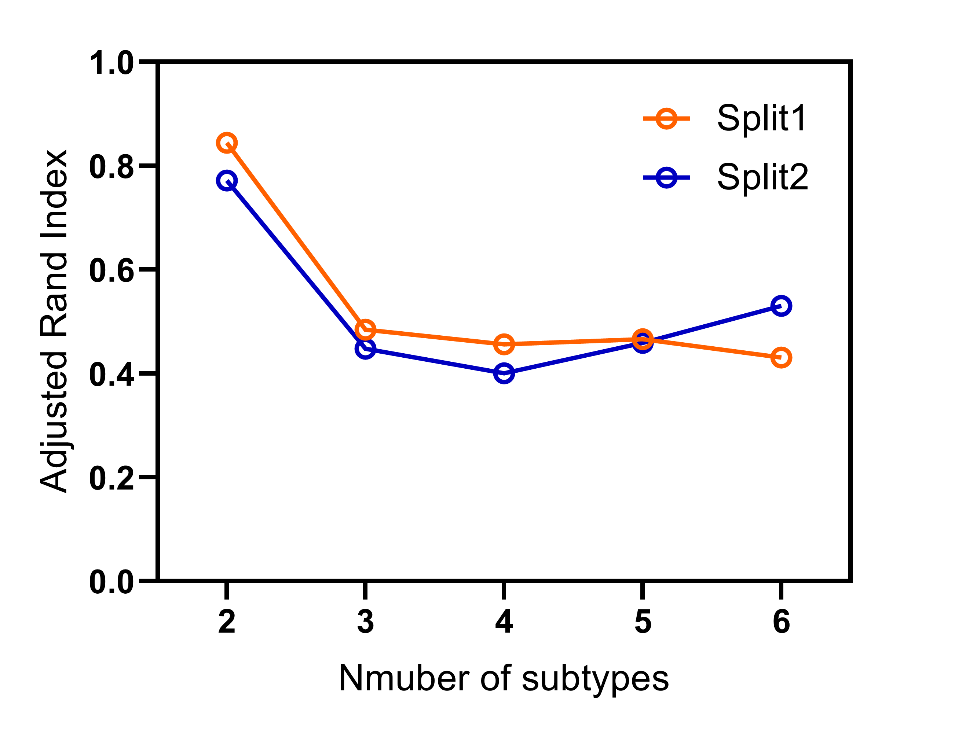


**Figure S2** The stability of the split-half samples was validated through cross-validation. Findings show that when K equals 2, the reproducibility of subtypes is consistently high across both split 1 and split 2.


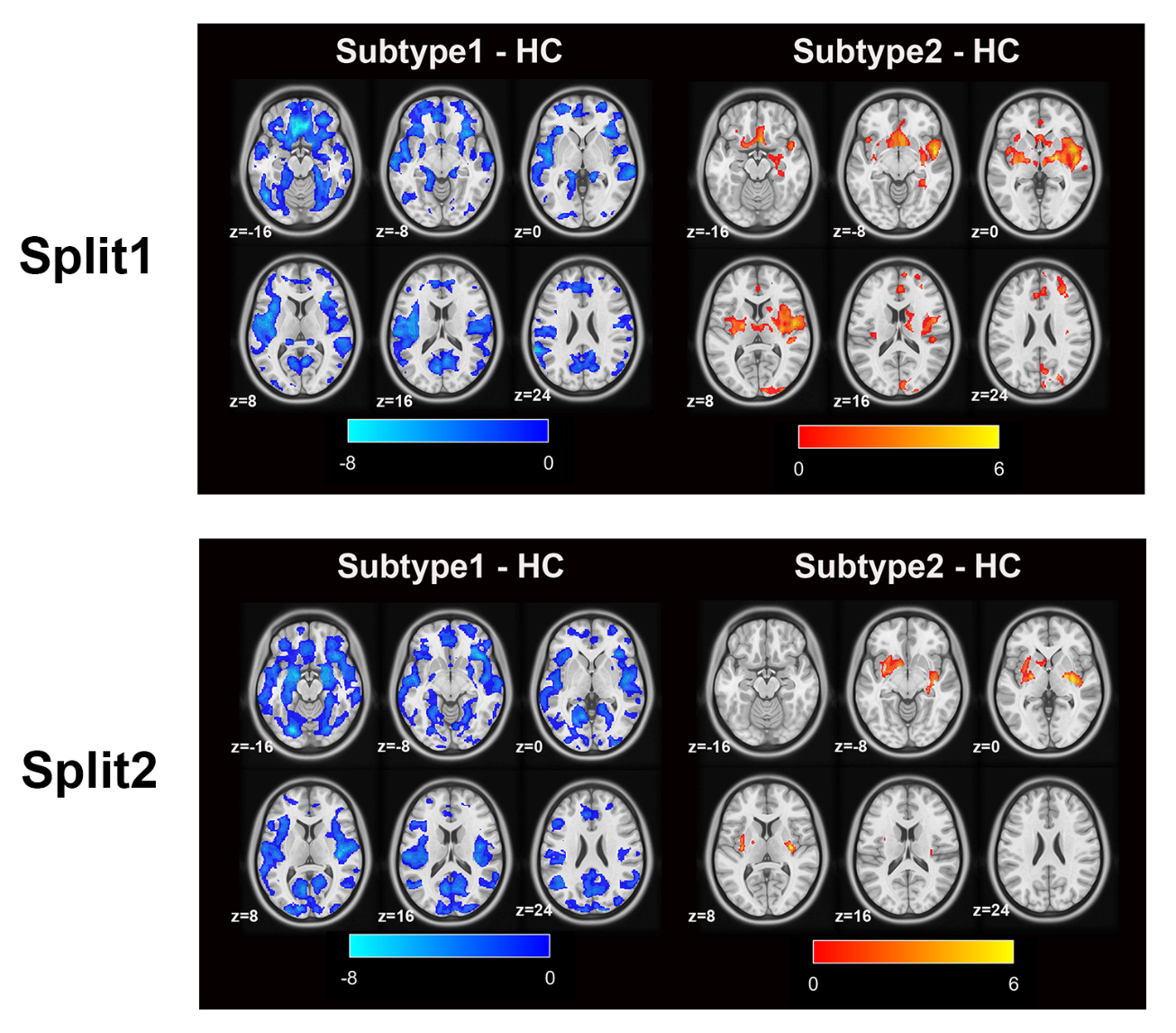


**Figure S3** GMV differences between each subtype and HC for K = 2 in Split 1 and Split 2. In both splits, the GMV patterns are consistent with those obtained from the entire sample, albeit weaker, largely due to the smaller sample size. (GRF correction, voxel p <0.001, cluster p <0.01)

1. Hentz JG, Mehta SH, Shill HA, Driver-Dunckley E, Beach TG, Adler CH. Simplified conversion method for unified Parkinson's disease rating scale motor examinations. *Mov Disord.* 2015;30(14):1967-1970.
